# A Multimodal CMOS Readout IC for SWIR Image Sensors with Dual-Mode BDI/DI Pixels and Column-Parallel Two-Step Single-Slope ADC

**DOI:** 10.3390/mi16070773

**Published:** 2025-06-30

**Authors:** Yuyan Zhang, Zhifeng Chen, Yaguang Yang, Huangwei Chen, Jie Gao, Zhichao Zhang, Chengying Chen

**Affiliations:** 1 School of Opto-Electronic and Communication Engineering, Xiamen University of Technology, Xiamen 361024, China; zhangyuyan@stu.pku.edu.cn (Y.Z.); chenhuangwei2178@163.com (H.C.); gaojie19981125@163.com (J.G.); zhichaozhang0605@163.com (Z.Z.); 2 School of Electronics, Peking University, Beijing 100871, China; 3 Health Electronics Center, Institute of Microelectronics (IME) of the Chinese Academy of Sciences, Beijing 100871, China

**Keywords:** CMOS readout integrated circuit (ROIC), buffered direct injection (BDI), direct injection (DI), short-wave infrared (SWIR), two-step single-slope ADC (TS-SS ADC)

## Abstract

This paper proposes a dual-mode CMOS analog front-end (AFE) circuit for short-wave infrared (SWIR) image sensors, which integrates a hybrid readout circuit (ROIC) and a 12-bit two-step single-slope analog-to-digital converter (TS-SS ADC). The ROIC dynamically switches between buffered-direct-injection (BDI) and direct-injection (DI) modes, thus balancing injection efficiency against power consumption. While the DI structure offers simplicity and low power, it suffers from unstable biasing and reduced injection efficiency under high background currents. Conversely, the BDI structure enhances injection efficiency and bias stability via an input buffer but incurs higher power consumption. To address this trade-off, a dual-mode injection architecture with mode-switching transistors is implemented. Mode selection is executed in-pixel via a low-leakage transmission gate and coordinated by the column timing controller, enabling low-current pixels to operate in low-noise BDI mode, whereas high-current pixels revert to the low-power DI mode. The TS-SS ADC employs a four-terminal comparator and dynamic reference voltage compensation to mitigate charge leakage and offset, which improves signal-to-noise ratio (SNR) and linearity. The prototype occupies 2.1 mm × 2.88 mm in a 0.18 µm CMOS process and serves a 64 × 64 array. The AFE achieves a dynamic range of 75.58 dB, noise of 249.42 μV, and 81.04 mW power consumption.

## 1. Introduction

Infrared focal plane arrays (IRFPAs) are pivotal in advanced photodetection systems for night vision and thermal imaging [1,2,3,4,5,6]. As the interface between infrared detectors and signal processors, the ROIC critically determines imaging quality through its charge-handling capability, noise performance, and dynamic range [7]. Although DI and BDI injection circuits have been extensively studied, their inherent performance trade-offs remain a central challenge for next-generation IRFPAs.

Recent high-resolution SWIR ROICs based on CTIA or digital front-ends underscore the demand for low-noise, high-throughput ADCs [8,9,10]. Emerging focal-plane arrays based on novel semiconductor detectors require a front-end that can accommodate both ultralow dark current (<1 nA) and large photocurrent (>20 nA). Within ROIC architectures, pixel-level current injection methods significantly affect signal fidelity and detector bias stability. The DI [11] approach, characterized by its minimalist structure, is advantageous in terms of compactness and ultra-low power consumption. However, its performance rapidly degrades under large photocurrents or variable illumination conditions due to insufficient injection efficiency and susceptibility to bias drift arising from impedance mismatch between the detector and the integration node. By contrast, the BDI [12] topology integrates a local buffer—typically a source follower or an operational amplifier—to decouple the detector from the integrator. This configuration substantially improves injection efficiency and bias stability, thereby enabling superior linearity and noise performance. Nevertheless, the inclusion of an active buffer incurs significant penalties in area and static power, which limits scalability, especially in large-format, power-constrained sensor arrays [13,14].

Concurrently, the analog-to-digital conversion stage imposes another critical performance bottleneck. The chip-level ADCs exhibit lower power consumption but are hindered by slow quantization speeds, making them unsuitable for high-frame-rate scenarios. Column-level ADCs strike a balance by combining the resolution advantages of pixel-level designs with the speed and power efficiency of chip-level implementations [15]. Therefore, column-parallel ADCs have become the de facto standard in high-resolution IRFPA architectures, balancing throughput and power efficiency. Successive-approximation-register (SAR) ADCs have achieved up to 14-bit resolution within 2 µs [16]. However, the large capacitor-DAC (CDAC) array incurs considerable area overhead, rendering SAR ADCs impractical for ultra-large-format sensors. Recent work has explored alternative architectures. Zhao et al. demonstrated a 12-bit two-stage cyclic ADC in 110 nm CMOS achieving 11.4 bit ENOB at 1 MS/s with only 0.185 mW per column (Walden FoM = 68.5 fJ/step) [17]. Cheng et al. reported a 4th-order noise-shaping SAR ADC in 28 nm CMOS yielding 94.3 dB SNDR over 100 kHz bandwidth and a Schreier FoM of 184 dB at 5 MS/s for just 107.4 µW [18]. Catania et al. further showed that inverter-based ΔΣ modulation can achieve >12 ENOB and 79.4 dB SNR at an ultra-low 71.5 nW power budget for wearable sensors [19]. While these approaches push FoM and resolution limits, they require multiple high-speed amplifiers, large capacitor networks, or complex feedback loops that constrain pixel pitch and complicate large-scale integration. Among available ADC designs, single-slope (SS) ADCs are notable for their architectural simplicity and inherent linearity. Compared to SAR and cyclic ADCs, SS ADCs significantly reduce column fixed-pattern noise (CFPN) by leveraging uniform ramp signals across all columns [20,21,22].

However, their serial quantization process imposes a substantial speed constraint—requiring 2N clock cycles for N-bit resolution—which is inadequate for high-frame-rate imaging. To mitigate this limitation, hybrid two-step single-slope (TS-SS) ADCs have been introduced. These converters decouple coarse and fine quantization, effectively reducing the number of required clock cycles to 2^M^ + 2^N^ for M + N bit resolution. Despite their improved speed, conventional TS-SS architectures suffer from critical non-idealities, including parasitic charge leakage and comparator offset, particularly during the fine quantization phase. These effects can significantly degrade signal-to-noise ratio (SNR), increase differential nonlinearity (DNL), and undermine conversion accuracy under low signal or high-speed conditions.

In response to these challenges, this work proposes a high-performance dual-mode AFE architecture tailored for SWIR image sensors. The design features a dynamically reconfigurable DI/BDI pixel interface that adapts to varying photocurrent conditions to optimize trade-offs between power consumption and injection efficiency. In parallel, a precision-optimized 12-bit TS-SS ADC is developed, incorporating a novel four-terminal comparator and dynamic reference voltage compensation. This approach effectively suppresses charge leakage and improves fine-ramp linearity, resulting in enhanced conversion fidelity. Fabricated in a standard 0.18 μm CMOS process, the proposed AFE achieves a measured dynamic range of 75.58 dB and power consumption of 81.04 mW. The ADC achieves an effective number of bits (ENOB) of 6.12 and a spurious-free dynamic range (SFDR) of 48.26 dB at 4 MS/s. These results demonstrate the proposed architecture’s suitability for high-sensitivity, low-power imaging systems operating in complex and dynamic environments.

## 2. System Architecture

**Figure 1:**
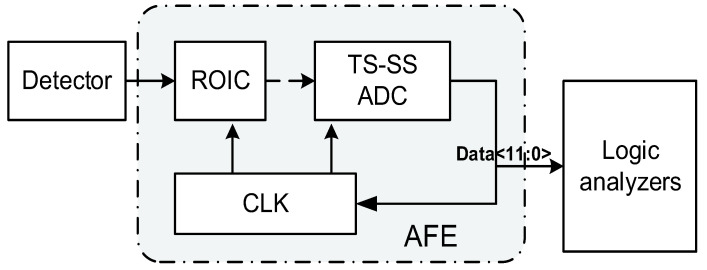
System-level block diagram of the SWIR AFE comprising the pixel array, DI/BDI front-end, timing controller, and TS-SS ADC.

**Figure 2:**
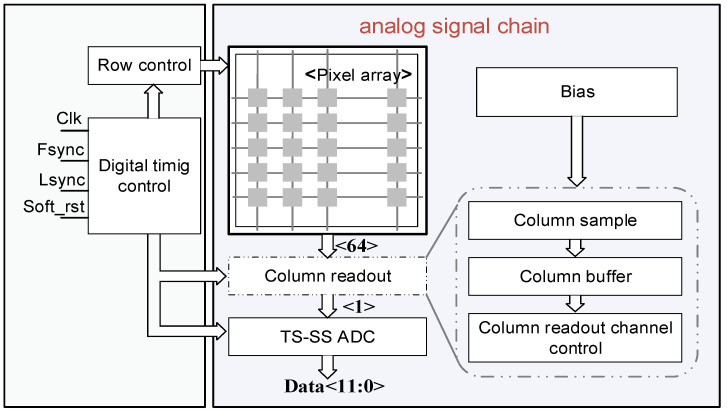
The analog front-end circuit framework.

Figure 1 gives a system-level view of the AFE, timing controller, and column ADC. Photocurrent from the pixel feeds a DI/BDI front-end, whose output is sampled by a two-phase track-and-hold (T/H). A global timing controller broadcasts coarse-/fine-ramp edges while locally gating column clocks to overlap integration with conversion. A 12-bit TS-SS ADC digitizes the held voltage and streams the data to a column serializer. The proposed AFE architecture is designed to support high-sensitivity, low-noise readout and digitization of short-wave infrared (SWIR) signals, as shown in Figure 2.

### 2.1. Analog Front-End with Dual-Mode

**Figure 3:**
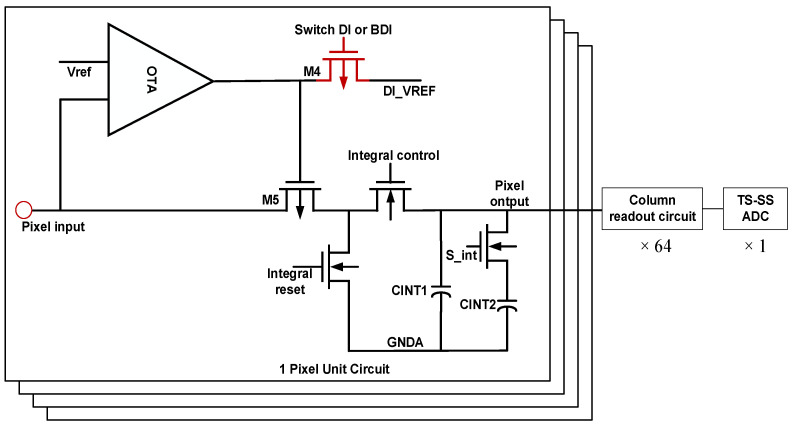
Dual-mode injection circuit diagram.

The AFE includes a dual-mode pixel array, column-level sample-and-hold (S/H) circuits, output buffers, and a column-parallel TS-SS ADC. As shown in Figure 3, each pixel features a reconfigurable injection front-end capable of toggling between DI and BDI modes. Unlike the CdS/CdTe neutron-detector model with poly-Si TFT amplifiers [23], the HGFET pixel offers gate-controlled gain and sub-nA dark current, necessitating a 10^3^ dynamic range without per-pixel gain switching [21]. Dual-mode injection is the best choice. The selection is dynamically controlled through a dedicated mode-switching transistor embedded at the pixel level. In DI mode, the photocurrent generated by the detector is directly integrated onto the storage capacitor. This configuration minimizes static power consumption and is suitable for low-light or power-constrained applications. In contrast, BDI mode activates a local buffer—typically implemented as an operational amplifier—to decouple the detector from the integration node. This enhances bias voltage stability and significantly improves injection efficiency under high background illumination or fluctuating signal levels. Following pixel-level integration, analog voltages are transferred onto column-level capacitors through row selection, then buffered by source followers to preserve signal fidelity during readout. These buffered voltages are subsequently digitized by the TS-SS ADCs located at the bottom of each column.

### 2.2. TS-SS ADC Design

**Figure 4:**
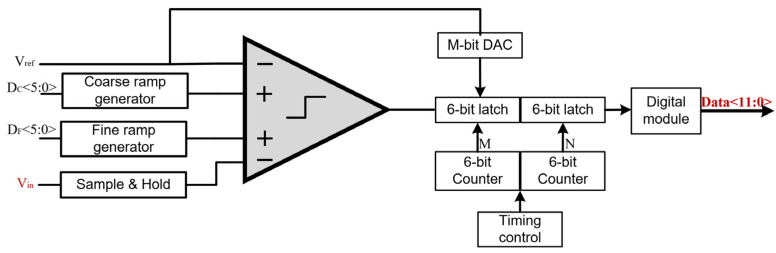
The diagram of the TS-SS ADC.

The column-parallel ADC structure integrates several key components: an S&H unit, a four-terminal comparator, coarse and fine ramp generators, and digital calibration logic, as shown in Figure 4. A central innovation of the ADC is the incorporation of a four-terminal comparator architecture, which mitigates the effects of parasitic charge leakage during the fine quantization phase. Conventional TS-SS ADCs typically connect the top plate of the storage capacitor to the fine ramp generator. This introduces parasitic capacitance that interacts with the held charge, leading to ramp distortion, degraded linearity, and increased signal-dependent errors. In the proposed design, the bottom plate of the storage capacitor is instead tied to ground, effectively decoupling the ramp generator from the capacitor node. This approach eliminates the dominant leakage path, ensuring consistent ramp slope and improving SNR.

### 2.3. Digital Timing Control

**Figure 5:**
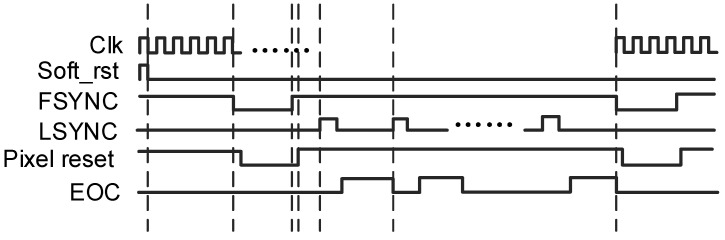
Digital timing control.

The digital module, synchronized to an external 20-MHz master clock, row sync (LSYNC), and frame sync (FSYNC) signals, generates precision timing signals, as shown in Figure 5. Triggered by three external signals—master CLK, LSYNC, and FSYNC—the digital module generates the timing control signal required by the readout circuit. These inputs govern the pixel selection sequence, ADC conversion cycles, and data readout windows. The controller generates phase-aligned clock signals for pixel integration, sample-and-hold activation, ramp generation, and digital readout. All control signals are passed through level shifters to ensure compatibility between digital logic and analog voltage domains. The system also supports programmable integration time and region-of-interest (ROI) windowing, allowing for flexible operation under varying scene dynamics and application-specific requirements.

## 3. Silicon-Based ROIC Design

The ROIC plays a pivotal role in bridging the interface between the detector array and the ADC, thereby determining the system’s noise floor, linearity, and signal integrity. To support adaptive performance across a wide dynamic range and varying illumination conditions, the proposed ROIC employs a dual-mode injection front-end architecture, capable of dynamically switching between DI and BDI modes at the pixel level.

### 3.1. Dual-Mode Pixel Injection Structure

Each pixel incorporates a reconfigurable input stage consisting of a photodetector, an integration capacitor, and a mode-switching transistor that determines the operational mode of the pixel. When operating in DI mode, the photocurrent generated by the detector is directly integrated onto the capacitor without any intermediate amplification. This mode is particularly advantageous in low-light scenarios or systems with stringent power budgets, as it minimizes the static power consumption and area overhead by eliminating active elements. In contrast, BDI mode leverages a local feedback amplifier to buffer the detector node from the integration capacitor. The op-amp maintains the detector bias at a constant voltage while enabling more efficient current injection through the main integrating transistor. The op-amp amplifies source-node voltage fluctuations by a factor of (Av + 1), effectively enhancing the transconductance (gm) of injection transistor M5. This configuration enhances the pixel’s linearity and suppresses signal-dependent bias variation, thereby improving the system’s dynamic performance in high-illumination or fast-transient environments. The transition between DI and BDI modes is governed by a single transistor (M4), which toggles the connection between the buffer amplifier and the pixel front-end. This transistor-based control scheme ensures seamless, real-time reconfiguration of the injection structure without the need for external reprogramming or power cycling.

### 3.2. Transconductance and Injection Efficiency

**Figure 6:**
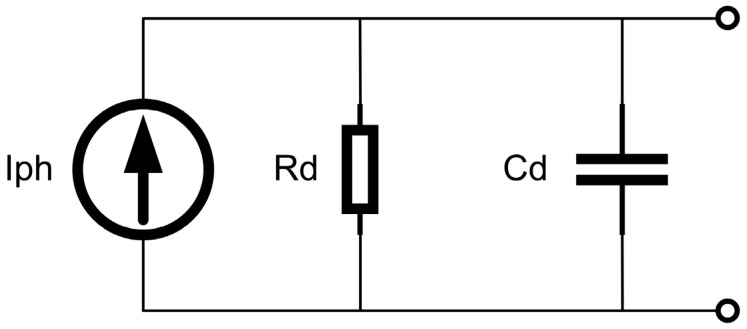
Small-Signal Model of the Photodetector.

To quantitatively analyze the performance advantages offered by the dual-mode architecture, the photodetector is modeled as a parallel network comprising a current source (I_ph_), an equivalent capacitance (C_d_), and a conductance (g_ds_), as shown in Figure 6. The photocurrent is routed through a MOS injection transistor (M_5_), whose transconductance (g_m1_) governs the efficiency of signal injection. The behavior of g_m1_ depends strongly on the transistor’s operating regime. When M5 operates in saturation, its transconductance is governed by:

(1) $$g_{msat}=\mu_{n}C_{ox}\frac{W}{L}(V_{GS}-V_{TH})$$

where *µn* is the electron mobility, C ox is the gate oxide capacitance, and W/L is the transistor aspect ratio. In subthreshold operation, gm exhibits distinct behavior due to exponential current-voltage relationships:

(2) $$g_{msub}=\frac{\mu_{n}C_{ox}\frac{W}{L}\frac{KT}{q}}{\left(V_{GS}-V_{TH}\right)}$$

Here, k is Boltzmann’s constant, T is the absolute temperature, and q is the electron charge. Subthreshold operation enhances transconductance efficiency, reducing sensor bias fluctuations (<0.1% nonuniformity error) and improving sensitivity.

- Injection Efficiency in BDI/DI Modes:

Injection efficiency (Y) is defined as the ratio of the effective integrated photocurrent (_Iint_) to the total generated photocurrent (I_ph_), serving as a critical metric for evaluating the front-end’s charge transfer performance. In DI mode, the injection efficiency is limited by the detector’s output impedance and is approximately expressed as:

(3) $$Y_{DI}=\frac{g_{m1}}{g_{m1}+g_{ds}}$$

where g_ds_ is the detector conductance. In BDI mode, the presence of the feedback amplifier effectively multiplies the transconductance by the amplifier gain A_v_, leading to an improved efficiency model:

(4) $$Y_{BDI}=\frac{(A_{v}+1)g_{m1}}{(A_{v}+1)g_{m1}+g_{ds}}$$

Here, A_v_ is the op-amp voltage gain. This relationship indicates that as A_v_ increases, the injection efficiency asymptotically approaches unity.

## 4. Silicon-Based ADC Design

The performance of the ADC plays a decisive role in determining the overall signal fidelity of infrared image sensors, particularly in systems requiring high frame rates and wide dynamic range. To address the latency and non-idealities inherent in conventional single-slope architectures, a column-parallel TS-SS ADC is proposed, incorporating architectural enhancements that significantly improve resolution, linearity, and energy efficiency.

### 4.1. Four-Terminal Comparator Architecture

We compared three comparator architectures—a standard op-amp-based design, a dynamic latch, and our implemented Differential Difference Amplifier (DDA). Pure op-amp comparators suffer from elevated 1/f noise under low SWIR photocurrents and require extensive per-column offset trimming, while dynamic latches introduce dead-time for offset-cancellation cycles, reducing throughput. The DDA, however, inherently suppresses column-to-column common-mode offset without additional calibration steps and maintains low input-referred noise, making it the optimal choice for large-scale SWIR focal planes.

**Figure 7:**
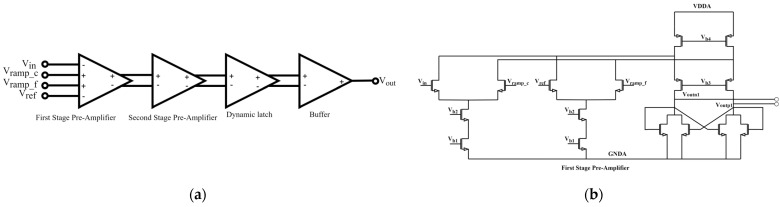
(**a**) Structure diagram of the four-terminal comparator; (**b**) Schematic of the first state amplifier.

After the charge leakage of the storage capacitor, the slope of the fine slope signal will be reduced, and a quantization dead zone will be generated. In more serious cases, the comparator may fail to trigger in the fine quantization stage. To suppress this issue, the proposed ADC incorporates a four-terminal comparator, wherein the bottom plate of the sampling capacitor is tied to GND, rather than connected to the fine ramp, as shown in Figure 7a. This architectural decoupling eliminates the primary leakage path through the parasitic capacitance and ensures stable voltage retention throughout the quantization period. The comparator comprises two stages:

- First Stage—Differential Difference Amplifier: Provides high open-loop gain and common-mode rejection, enabling accurate resolution of small voltage differences during both coarse and fine quantization. Figure 7b depicts a partial transistor-level schematic of the four-terminal dynamic comparator. The core employs a cross-coupled NMOS latch with PMOS active loads. Two auxiliary switches momentarily tie the storage capacitor C_H_ to the latch tail during reset, canceling kick-back.
- Second Stage—Diode-Loaded Inverter: Offers high-speed signal regeneration with minimal propagation delay, ensuring rapid latching behavior at the threshold crossing.

By structurally isolating the ramp signal from the storage node and incorporating a precision amplifier front-end, the comparator exhibits superior sensitivity and immunity to charge injection artifacts.

### 4.2. Quantization Scheme with Redundant Bit Allocation

**Figure 8:**
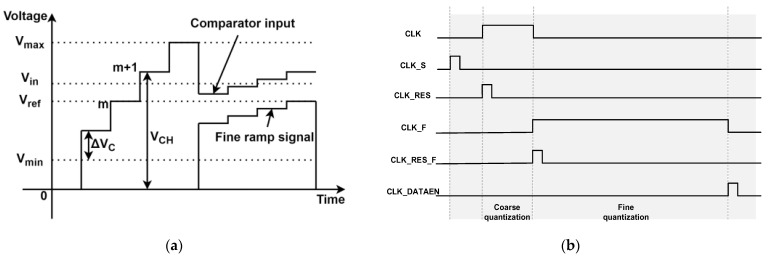
(**a**) The relationship between the signals; (**b**) Timing design.

To overcome nonlinearity introduced by device mismatches and transition glitches, a hybrid quantization algorithm is adopted. The total 12-bit resolution is achieved through a coarse-fine division of 6 bits each, supplemented by a 1-bit redundancy mechanism that facilitates digital post-correction. Figure 8a shows our two-step conversion. First, a 6-bit coarse counter generates a global ramp and latches the MSBs. The residue amplifier then subtracts this coarse voltage from the input. Next, a 6-bit fine ramp—extended with one redundant bit—captures the LSBs. Finally, digital correction logic aligns MSB and LSB codes, removing any ±1 LSB offset via the redundant bit to produce the 12-bit output.

**Figure 9:**
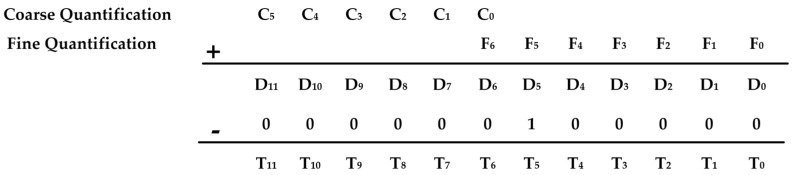
Schematic diagram of quantization result processing.

The quantization process proceeds as follows:

- Coarse Phase: A 6-bit counter generates a globally distributed voltage ramp, enabling parallel acquisition of the most significant bits (MSBs) across all columns. This ramp resolves the voltage to a 64-level approximation.
- Fine Phase: A higher-resolution ramp is applied to the fine quantization stage, covering a dynamic range of 2ΔV to fully span the residue from the coarse stage. To account for transition errors, the fine ramp includes one extra redundant bit.
- Digital Correction: Post-processing logic combines the coarse and fine quantization results. As shown in Figure 9, an adder aligns the fine MSB with the coarse result, and a subtractor removes a fixed digital offset introduced by the redundancy, yielding a final 12-bit output with minimized DNL.
- Simulation and silicon measurements demonstrate that the proposed scheme suppresses non-monotonicity and reduces worst-case DNL to below 0.2 least significant bits (LSB), even in the presence of offset voltage mismatch and charge redistribution.

### 4.3. Switch Charge Injection Compensation

Switch charge injection is a dominant source of nonlinearity in TS-SS ADCs. When switches open during coarse quantization, the injected charge (Q_inj_) alters the voltage on C_H_:

(5) $$Q_{inj}=\delta (WL{)}_{S1}C_{ox}(V_{GS}-V_{TH})=\delta (WL{)}_{S1}C_{ox}(V_{DD}-(M+1)\Delta V-V_{TH})$$

where δ is the charge injection ratio, (WL) S1 is the switch transistor dimensions, and M represents the coarse quantization result. This charge induces a voltage shift (ΔV_CH_) on C_H_:

(6) $$\Delta V_{CH}=\frac{Q_{inj}}{C_{H}}=\frac{\delta (WL{)}_{S1}C_{ox}(V_{DD}-(M+1)\Delta V-V_{TH})}{C_{H}}$$

To counteract this perturbation, a 6-bit current-steering digital-to-analog converter (DAC) is introduced to adjust the reference voltage Vref dynamically. This compensation voltage aligns the comparator decision threshold with the expected ramp trajectory:

(7) $$V_{DAC(m)}=\Delta V_{CH}(m)+V_{ref}$$

This adjustment ensures the comparator input remains linear:

(8) $$V_{in}=m\Delta V-VS-\frac{1}{2}\Delta V+i\Delta F$$

Through this scheme, the static and dynamic characteristics of the TS-SS ADC can be effectively improved to ensure the high precision and stability of the analog-to-digital conversion. Figure 8a shows the relationship between the input signal Vin, the coarse ramp voltage, the fine ramp voltage and the storage capacitor ΔV_CH_ during the quantization process.

### 4.4. ADC Timing and Control Strategy

The ADC operates in three phases, synchronized to a 20-MHz master clock as shown in Figure 8b:

- Sampling Phase: The pixel output voltage is sampled and held (CLK_S high);
- Coarse Quantization Phase: The 6-bit counter generates V_Coarse_ (CLK high), resolving MSBs within 64 cycles;
- Fine Quantization Phase: A 7-bit counter drives V_fine_ (CLK_F high), with redundancy and DAC compensation refining the least significant bits (LSBs);
- All transitions are synchronized to a 20 MHz master clock and governed by global synchronization signals (LSYNC, FSYNC), ensuring deterministic latency and pipeline compatibility. The entire column-parallel array can thus operate in parallel without temporal skew, enabling real-time readout of high-resolution frames. A foreground redundancy-based calibration removes coarse-stage transition errors, while the on-chip PLL offers background time-base calibration that trims the fine-ramp slope to <0.05% error.

### 4.5. Charge-Injection Compensation DAC

The switch network that connects the coarse-stage residue to the fine-ramp capacitor injects ± charge when turning off, producing a deterministic staircase on the fine ramp and degrading INL by up to 0.7 LSB. To cancel this error, a 6-bit DAC sinks a programmable bias current from the fine-ramp buffer. During reset, the same switches are held closed so the injected charge is mirrored into the DAC sink node; by trimming the DAC code, the net charge ∆Q is nulled.

## 5. Experimental Results

**Figure 10:**
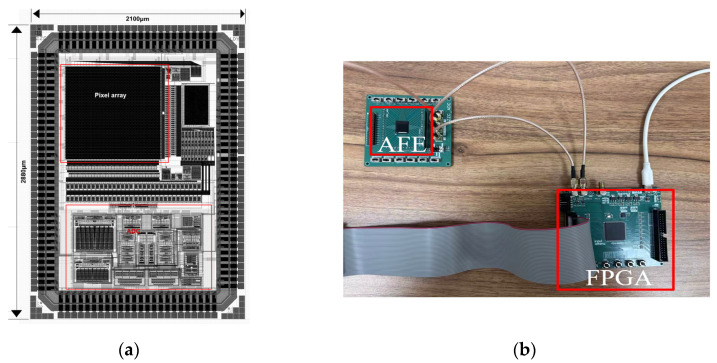
(**a**) The layout of the AFE; (**b**) the test system.

The proposed dual-mode AFE was fabricated in a standard 180 nm 1P7M CMOS process. The layout is shown in Figure 10a, with an area of 2100 μm × 2880 μm. The silicon prototype includes a 64 × 64 pixel array with reconfigurable DI/BDI pixel front-end, column-level TS-SS ADCs, and supporting digital control logic. As shown in Figure 10b, all measurements were conducted at room temperature (25 °C) under a 3.3 V supply, using a programmable logic analyzer and a high-precision current source to emulate detector output currents. A programmable low-noise current source delivered photocurrent replicas from 100 pA to 100 nA based on a Verilog-A SWIR photodetector model extracted from Ref. [24]. In some experimental tests, an external resistor array was used to simulate the current input of the detector. Output data (12-bit × 64 columns) were captured through LVDS pairs into a TLA6402 Logic Analyzer and de-skewed in MATLAB (Version: 9.13.0 (R2022b)) for FFT and code-density analysis. No photodetector array was bonded in this tape-out; full electro-optical tests are planned for the second silicon revision. It should be noted that the evaluation board was a first-revision four-layer design aimed at rapid silicon bring-up rather than optimum analog performance. The open bench-top environment exhibited a −62 dB spur due to ambient mains hum.

### 5.1. ROIC Performance

**Figure 11:**
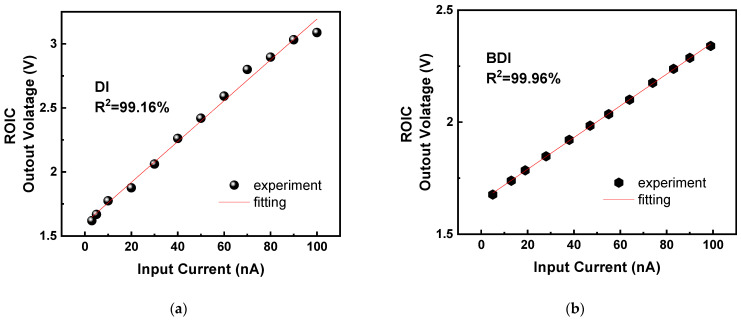
(**a**) BDI linearity; (**b**) DI linearity.

To assess the effectiveness of the dual-mode readout structure, we first performed isolated measurements on the ROIC by bypassing the ADC. A linear ramp current input ranging from 0.1 nA to 100 nA was applied, and the output voltage was recorded for both DI and BDI configurations, as shown in Figure 11a,b. In BDI mode, the ROIC demonstrated a maximum linearity of 99.56%, compared to 98.7% in DI mode. This improvement is directly attributed to the buffer amplifier’s ability to maintain a constant detector bias and decouple signal-dependent impedance variations. Moreover, the feedback mechanism in BDI significantly reduces nonlinearity under large-signal operation by preserving the integrity of the integration node potential. These results confirm that BDI mode is more suitable for high dynamic range or bright-scene conditions, while DI mode offers acceptable linearity at significantly reduced power consumption, making it preferable in low-illumination, power-constrained scenarios.

### 5.2. ADC Performance Evaluation

**Figure 12:**
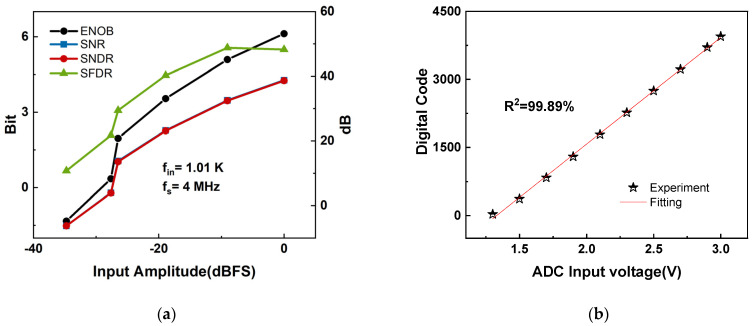
(**a**) Measured output spectrum, ENOB/SNR/SNDR/SFDR versus input frequency; (**b**) the relationship between the input voltage signal ADIN of the ADC and the integrated voltage output.

Figure 12a demonstrates that the TS-SS ADC attains 6.12-bit ENOB and 48.26 dB SFDR at 4 MHz. Three dominant error sources were identified:

- Residual fine-ramp distortion due to second-order parasitic leakage around CH even after the four-terminal comparator decoupling, shortening the effective LSB range and degrading SNR.
- Clock-edge jitter of 18 ps rms propagates to 0.55 LSB timing uncertainty during the 7-bit fine ramp, removing ~0.4 bits.
- Board-level parasitics further reduce the measured ENOB: (i) supply noise contributes 0.6 bits per IEEE Std-1241 SNR scaling; (ii) ramp-line capacitance increases the effective LSB by 0.5 LSB. These losses are extrinsic and will be mitigated by a six-layer PCB with isolated analog grounds, on-board LDO filtering, differential ramp routing, and an aluminum enclosure. The output digital code of the ADC was tested with an incremental voltage step of 0.1 V. Each voltage generates 4096 digital codes, and the average value of the digital codes is taken to fit the line that coincides with the scatter plot. The four-terminal comparator eliminates charge leakage, achieving 99.89% linear ramp slope accuracy, as shown in Figure 12b. The architecture achieves 4220 fJ/conv-step (ADC-core) while offering dual-mode BDI/DI flexibility at the system level.

### 5.3. Pre- vs. Post-Layout Simulation Correlation

Table 1 overlays ADC spectra from schematic-level (pre-layout), RC-extracted post-layout (PEX) netlists, and measured silicon in BDI mode. Table 1 quantifies ENOB, SFDR and SNDR. Post-layout parasitics degrade ENOB by 0.12 bit, attributable to additional routing capacitance. The extra 5.63-bit drop from PEX to silicon stems from PCB supply noise, clock jitter, comparator kick-back underestimated in PEX, and package bond wire inductance.

### 5.4. Comparative Analysis

**Figure 13:**
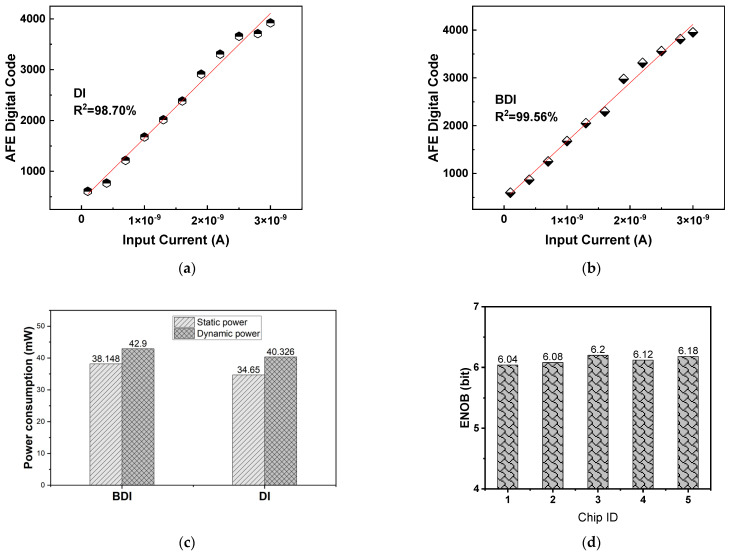
(**a**) The relationship between the input current signal and the output digital code of the AFE in DI mode; (**b**) the relationship between the input current signal and the output digital code of the AFE in BDI mode; (**c**) power consumption of BDI and DI modes; (**d**) measured ENOB versus die.

Finally, the dynamic range of the AFE was tested, and the test results are shown in Figure 13a,b. Both AFE chips in BDI and DI modes can achieve signal acquisition and processing of detector arrays. In DI mode, the AFE exhibits 98.7% linearity, whereas BDI mode attains 99.56%, owing to the buffer’s ability to stabilize detector bias. As shown in Figure 13c, measured power consumption under different operating conditions is summarized as follows:

- DI Mode: 74.98 mW (static + dynamic);
- BDI Mode: 81.04 mW (≈8% increase over DI).

The additional power in BDI mode is attributable to the buffer amplifier and associated bias circuits. Nonetheless, this modest increase is justified by significant improvements in injection efficiency, bias stability, and readout linearity. The trade-off capability between power and performance highlights the effectiveness of the proposed dual-mode structure in dynamic or mission-adaptive imaging systems. Designers can select DI or BDI mode in real time based on ambient light level or system power state. We have measured five dies from the same wafer lot. Figure 13d shows the ENOB (6.12 ± 0.08 bit). The tight distributions (<1% variation) confirm robustness against process drift.

To further validate the competitiveness of the proposed AFE, a comprehensive comparison with prior art is presented in Table 2.

- Pixel Size: Among dual-mode AFEs, this work achieves the smallest pixel pitch (15 μm), enhancing spatial resolution and integration density;
- Injection Range: The widest current sensing range (100 pA to 100 nA) is achieved, supporting both ultra-low signal and high-flux scenarios;
- Linearity: Linearity in BDI mode (99.56%) matches or exceeds that of high-end fixed-mode ROICs, validating the efficacy of buffer-assisted injection;
- Power Efficiency: Despite its dual-mode capability and integrated 12-bit ADC, the power remains within the same order as single-mode designs, emphasizing circuit efficiency. The 74.976 mW/81.048 mW figure refers to the full dual-mode front-end (BDI + DI) plus column ADC array and on-chip timing controller, whereas Ref. [26] reports a BDI/DI front-end. Isolating our ADC core yields 0.01 mW/pixel; however, Ref. [28] reaches 0.12 mW/pitch.

## 6. Conclusions

This work presents a dual-mode CMOS analog front-end for image sensors, integrating a dynamically reconfigurable BDI/DI ROIC and a 12-bit TS-SS ADC. Key innovations include the following:

- Dual-Mode Pixel Architecture: The BDI/DI hybrid input stage enables dynamic switching between high injection efficiency (99.56% at 100 nA) and low power consumption (74.96 mW), achieving a 75.58 dB dynamic range in BDI mode and 8% power savings in DI mode. The feedback op-amp in BDI mode stabilizes detector bias voltage, reducing nonlinearity to <0.1% under varying photocurrent levels;
- Four-Terminal Comparator ADC: By grounding the storage capacitor’s bottom plate, charge leakage is eliminated, ensuring 99.95% linearity in the fine ramp signal;
- Dynamic reference-voltage compensation enables 48.3 dB SFDR and a measured 6.12-bit ENOB over a 100 pA–1 nA photocurrent range.

The proposed architecture is particularly suited for applications requiring high sensitivity and adaptability, such as night vision, medical imaging, and industrial inspection. Future work will focus on further optimizing high-speed quantization stability and integrating on-chip temperature compensation for harsh environments.

## Figures and Tables

**Table 1:** Performance comparison with pre-layout, RC-extracted post-layout (PEX) netlists, and measured silicon in BDI mode.

Parameter	Pre-Layout	Post-Layout	Silicon Data
**SFDR (dB)**	81.55	87.67	48.3
**SNDR (dB)**	73.26	73.14	38.65
**ENOB (bit)**	11.87	11.75	6.12

**Table 2:** Performance comparison with AFEs.

Parameter	[25]	[26]	[27]	[28]	[29]	[30]	This Work
**Process**	180 nm CMOS	350 nm CMOS	180 nm CMOS	180 nm CMOS	350 nm CMOS	180 nm CMOS	180 nm CMOS
**Pixel array**	640 × 512	10 × 8	640 × 512	32 × 32	128 × 128	200 × 200	64 × 64
**Pixel size (** **μ** **m^2^)**	15 × 15	30 × 30	20 × 20	22.5 × 22.5	50 × 50	5 × 5	15 × 15
**Supply (V)**	1.8	5	3.3/1.8	NA	NA	3.3/1.8	3.3
**Voltage Swing (V)**	1	2	<2	1.5	1.4	/	1.5
**Pixel input type**	BDI	DI/BDI	DI	SFD/CTIA	BDI	/	DI/BDI
**Input current (A)**	100f-100n	1p-10n	N/A	15p-3n	22n-112n	/	100p-100n
**Power (mW)**	N/A	9.94	N/A	N/A	N/A	0.108 (only column ADC)	74.976/81.048
**Linearity**	99.95%	98%/99%	N/A	98.9%	95.8%	/	98.7%/99.5%
**Area (mm** × **mm)**	11.13 × 8.78	/	15 × 15.6	/	/	/	2.1 × 2.88
**ADC type**	/	/	/	/	/	Column parallel SS ADC	TS-SS ADC
**ADC resolution (bit)**	/	/	/	/	/	11	6.12

## Data Availability

All data are reported/cited in the paper.

## References

[1] Yin X., Zhang C., Guo Y., Yang Y., Xing Y., Que W. (2021). PbS QD-Based Photodetectors: Future-Oriented Near-Infrared Detection Technology. J. Mater. Chem. C.

[2] Tao Y., Xiong S., Song R., Muller J.P. (2021). Towards streamlined single-image superresolution: Demonstration with 10 msentinel-2 colour and 10–60 m multi-spectral VNIR and SWIR bands. Remote Sens..

[3] Zhang W., Linga K., Evans M.J., Liobe J.C., Chang D.S., Huang W., Enriquez M., Bereznycky P., Endicter S., Morales M. (2021). Development of high-performance SWIR detectors for passive and active imaging applications[C]//Infrared Technology and Applications XLVII. Int. Soc. Opt. Photonics.

[4] Bluzer N., Stehlik R. (1978). Buffered direct injection of photocurrents into charge coupled devices. IEEE J. Solid State Circuits.

[5] Goulding F.S., Landis D.A. (1982). Signal processing for semiconductor detectors. IEEE Trans. Nucl. Sci..

[6] Li X., Gong H.-M., Shao X.-M., Li T., Huang S.-L., Ma Y.-J., Yang B., Zhu X.-L., Gu Y., Fang J.-X. (2022). Recent advances in short wavelength infrared InGaAs focal plane arrays. J. Infrared Millim. Waves.

[7] Ma W., Shi Y., Zhang Y., Wu Z. A high speed snap-shot mode readout circuit for QWIP IR FPAs. Proceedings of the 2009 4th IEEE Conference on Industrial Electronics and Applications.

[8] Poonnen T., Esparza K., McCotter S., Ratledge B., Korth W., Dhawan N., Veeder K. (2024). Low-Noise High-Sensitivity DROIC for 640 × 512 SWIR FPAs. SPIE Conference Proceedings.

[9] Maestro R.J., Merken P., Tavernier F. A > 70 dB Digital ROIC in 65 nm CMOS for 1 Mp SWIR. Proceedings of the IEEE Sensors Conference.

[10] Mu Y., Zhao Z., Chen C., Yuan D., Wang J., Gao H., Chi Y. (2023). The Design of a Low-Noise, High-Speed Readout-Integrated Circuit for Infrared Focal Plane Arrays. Sensors.

[11] Chatterjee A., Abhale A., Pendyala N., Rao K.S.R.K. (2020). Group II–VI semiconductor quantum dot heterojunction photodiode for mid wave infrared detection. Optoelectron. Lett..

[12] Zhou T., Liu J., Fu B., Cao Y., He Y., Jiang B., Su Y. (2019). A high-precision and high-linearity readout integrated circuit for infrared focal plane array applications. Optik.

[13] Hsieh C.-C., Wu C.-Y., Jih F.-W., Sun T.P. (1997). Focal-plane-arrays and CMOS readout techniques of infrared imaging systems. IEEE Trans. Circuits Syst. Video Technol..

[14] Hsieh C.-C., Wu C.-Y., Sun T.-P. (1997). A new cryogenic CMOS readout structure for infrared focal plane array. IEEE J. Solid State Circuits.

[15] El Gamal A., Eltoukhy H. (2005). CMOS image sensors. Circuits Devices Mag. IEEE.

[16] Kim J.B., Hong S.K., Kwon O.K. (2015). A Low-Power CMOS Image Sensor with Area-Efficient 14-bit Two-Step SA ADCs Using Pseudomultiple Sampling Method. IEEE Trans. Circuits Syst. II Express Briefs.

[17] Zhao S., Gao J., Chen Q., Nie K., Xu J. (2024). 12-bit 2.5-bit/phase two-stage cyclic ADC with phase scaling and low-power Sub-ADC for CMOS image sensor. Microelectron. J..

[18] Cheng K.-C., Chang S.-J., Chen C.-C., Hung S.-H. (2025). A 94.3-dB SNDR 184-dB FoMs 4th-Order Noise-Shaping SAR ADC With Dynamic-Amplifier-Assisted Cascaded Integrator. IEEE Solid State Circuits Lett..

[19] Catania A., Gagliardi F., Piotto M., Bruschi P., Dei M. (2024). Ultralow-Power Inverter-Based Delta-Sigma Modulator for Wearable Applications. IEEE Access.

[20] Furuta M., Nishikawa Y., Inoue T., Kawahito S. (2007). A High-Speed, High-Sensitivity Digital CMOS Image Sensor with a Global Shutter and 12-bit Column-Parallel Cyclic A/D Converters. IEEE J. Solid State Circuits.

[21] Bae J., Kim D., Ham S., Chae Y., Song M. (2014). A Two-Step A/D Conversion and Column Self-Calibration Technique for Low Noise CMOS Image Sensors. Sensors.

[22] Ling Y.H., Wei Y.H., Jun W. (2009). Design of high frame rate CMOS image acquisition system based on PCI Express. Appl. Electron. Tech..

[23] Hernandez-Gutierrez C.A., Avila-Avendano C., Solis-Cisneros H.I., Conde J., Sevilla-Camacho P.Y., Quevedo-Lopez M.A. (2022). Modeling and SPICE Simulation of the CdS/CdTe Neutron Detectors Integrated with Si-Poly TFTs Amplifiers. IEEE Trans. Nucl. Sci..

[24] Zhou S., Zhang X., Wang Y., Lin D., Zou S., Wang J., Xiao L., Zhang D., Jiang J., Zhang P. (2024). Opto-Electrical Decoupled Phototransistor for Starlight Detection. Adv. Mater..

[25] Li H., Hu A., Nie Z., Liu D., Niu G., Gao L., Tang J. (2022). A 640 × 512 ROIC with optimized BDI input stage and low power output buffer for CQDs-based infrared image sensor. Microelectron. J..

[26] Sun T.-P., Lu Y.-C., Kang L.-L., Shieh H.-L. (2014). A buffer direct injection and direct injection readout circuit with mode selection design for infrared focal plane arrays. Infrared Phys. Technol..

[27] Selim E., Samet I.O., Nusret B., Murat I., Soyer S.T., Ustundag C.M.B., Kocak S., Turan O., Eksi U., Akin T. (2016). A 640 × 512-20 um Dual-Polarity ROIC for MWIR and LWIR Hybrid FPAs. SPIE Defense + Security.

[28] Yazici M., Ceylan O., Shafique A., Abbasi S., Gurbuz Y. High Dynamic Range Smart Pixel Architecture for Infrared Focal Plane Arrays. Proceedings of the 2018 IEEE International Symposium on Circuits and Systems (ISCAS).

[29] Woo D.H., Nam I.K., Lee H.C. (2010). Smart reset control for wide-dynamic-range LWIR FPAs. IEEE Sens. J..

[30] Ikebe M., Uchida D., Take Y., Someya M., Chikuda S., Matsuyama K., Asai T., Kuroda T., Motomura M. Image Sensor/Digital Logic 3D Stacked Module Featuring Inductive Coupling Channels for High Speed/Low-Noise Image Transfer. Proceedings of the Symposia on VLSI Technology and Circuits.

